# Applying ethics to AI in the workplace: the design of a scorecard for Australian workplace health and safety

**DOI:** 10.1007/s00146-022-01460-9

**Published:** 2022-05-13

**Authors:** Andreas Cebulla, Zygmunt Szpak, Catherine Howell, Genevieve Knight, Sazzad Hussain

**Affiliations:** 1 Australian Industrial Transformation Institute, Flinders University, Adelaide, Australia; 2 Australian Institute for Machine Learning, University of Adelaide, Adelaide, Australia; 3 Independent Consultant, University of Adelaide, Adelaide, Australia; 4 South Australian Centre for Economic Studies, University of Adelaide, Adelaide, Australia; 5 Centre for Work Health and Safety, Parramatta, NSW Australia

**Keywords:** Ethics principles, Risk assessment, AI Canvas, WHS/OHS, Workers, Australia

## Abstract

Artificial Intelligence (AI) is taking centre stage in economic growth and business operations alike. Public discourse about the practical and ethical implications of AI has mainly focussed on the societal level. There is an emerging knowledge base on AI risks to human rights around data security and privacy concerns. A separate strand of work has highlighted the stresses of working in the gig economy. This prevailing focus on human rights and gig impacts has been at the expense of a closer look at how AI may be reshaping traditional workplace relations and, more specifically, workplace health and safety. To address this gap, we outline a conceptual model for developing an AI Work Health and Safety (WHS) Scorecard as a tool to assess and manage the potential risks and hazards to workers resulting from AI use in a workplace. A qualitative, practice-led research study of AI adopters was used to generate and test a novel list of potential AI risks to worker health and safety. Risks were identified after cross-referencing Australian AI Ethics Principles and Principles of Good Work Design with AI ideation, design and implementation stages captured by the AI Canvas, a framework otherwise used for assessing the commercial potential of AI to a business. The unique contribution of this research is the development of a novel matrix itemising currently known or anticipated risks to the WHS and ethical aspects at each AI adoption stage.

## Introduction

Artificial intelligence (AI) has given rise to a new type of machine, a prediction machine, which can automate cognitive tasks traditionally associated with white-collar workers. These smart machines are lowering the cost and effort of making accurate predictions in operational processes. The AI technology may thus have potential benefits, such as increasing productivity, streamlining processes, and integrating value chains. Advocates of AI maintain that companies that do not take advantage of these benefits risk being driven out of the marketplace by others that do. As human expertise is being outperformed by AI, companies may prefer delivering their products and services, internally as well as externally, with the help of AI. The impact that such operational and logistical change in the workplaces may have on workers, however, remains uncertain.

Sociologists of work have long argued that workplace technologies are a key instrument of social change, reshaping labour relations and introducing new systems of worker discipline and control (Barley 1988; Braverman 1974; Kellogg et al. 2020; Zuboff 1988). From this critical sociological perspective, AI technologies in the workplace represent a new, contested domain for workers, raising questions around job security, worker autonomy, and worker status for which there is yet no clear social consensus. There is emerging research evidence on the qualitative impacts of AI on workers in specific industries, particularly insecure workers in the gig economy (Myhill et al. 2020; Convery et al. 2020). While the COVID-19 pandemic of 2020 has, at least temporarily, normalized flexible and remote work, many firms had already integrated complex, networked technologies integrating AI even before the pandemic to deliver products and services across borders and time zones, and to deploy skilled workers in more effective ways. The rise of networked technologies and “digital first” business strategy played out in the global economic context of increasingly precarious forms of employment, unequal wage growth, the transformation of manufacturing, and a growing income and skills divide in the workforce leading to reduced economic and social mobility. These outcomes are not inevitable, but are the result of personal, economic and political decisions (Srnicek and Williams 2016; Benanav 2020). Whether the new technologies adversely affect the security and wellbeing of employees in a workplace is above all a question of whether they are permitted to do so, or whether instead these risks are contained by regulation and oversight.

Harm may arise from the psychosocial impacts of organisational change and the specific ways that introducing AI modify the tasks and responsibilities of certain job roles (e.g., IEEE 2016; OECD 2019; Australian Human Rights Commission 2019). Workers in companies engaging with AI may experience anxiety about the longevity of their jobs and may become deeply suspicious of the new AI technology. Workers may be concerned that the new AI technology deployment could “take their jobs”, or they may feel uncertain about the coming workplace changes, including the performance of the new technology and how this affects their own job roles. AI may not just complement but may also challenge conventional knowledge and expertise, and thus force its users to reconsider workplace routines or professional judgement (for an example from the medical professions, see Lebovitz et al. 2022). Machine predictions may prove wrong or inappropriate and conflict with an employee’s moral as well as professional judgement, but that employee may be afraid to speak out and contradict the machine, fearing the consequences. Furthermore, there are risks of greater work-related stress as a result of increased worker monitoring with AI that incorporates wearable and Internet of Things (IoT) technologies and sets blurred boundaries between work and private life. The research and practitioner communities recognise these risks, many of which fall under the auspices of workplace health and safety.

To the knowledge of the authors, there has to-date been no systematic attempt at exploring and presenting the *workplace* risks associated with an increased use of AI. Thus, this research sought to address this gap in risks of AI use in the workplace and safeguarding workers. Building on a review of a growing but still comparatively ‘thin’ literature on the topic and, hence, complemented by exploratory expert and practitioner interviews conducted in Australia, the study explored the risks and potential harms that the introduction of AI in a workplace may pose to workers using the technology or being subject to its use. This paper outlines the development of a novel risk assessment tool or ‘scorecard’ identifying potential workplace-related risks of AI from a work health and safety (WHS) perspective, as an entry point for raising awareness and influencing AI-safe work practices. In doing so, this research drew on Australian national AI Ethics Principles, endorsed by the Australian Government, and applied these to AI implementation strategies through a series of consultations with feedback loops to WHS principles as set out by Safe Work Australia (SWA), the regulatory body responsible for enforcing and improving safe working practices in workplaces. In consultation with AI experts, WHS professionals, regulators, policymakers as well as organisations adopting AI for logistical or other production or service-related matters (more detail below), the AI-related ethical principles corresponding to WHS schemata of risks and hazards associated with the characteristics of work were investigated and incorporated into the proposed scorecard.

The rest of the paper is structured as follows. We commence with a brief background of known workplace risks of AI and how existing WHS practices interact with prominent identified AI risks. We then set out the proposition that Human Dignity is an essential foundation for framing AI risks in the workplace, which allows AI ethics frameworks to establish human centred AI development. We then evidence a conceptual linkage which shifts these from abstract concepts to a materially practical risk assessment tool. We include detail of the development process and how this formulation has been validated through the practice-led consultation approach. In concluding, we present the current status of our proposed risk assessment tool and its potential in helping companies adopt AI solutions while championing the health and safety of workers. We note that reframing ethics as a work health and safety issue has the benefit of using language that will be familiar to organisations from their existing health and safety audit and compliance processes, potentially reducing organisational resistance to introducing new processes for AI risk management.

## Organisations underappreciate workplace risks of AI

Public and academic debate is concerned about the potential impact of AI on labour markets or, more specifically, the extent to which AI may lead to the de-skilling of the workforce. But as Paschen et al. (2020) have demonstrated, AI adoption by employers can have alternative outcomes than de-skilling and may be competence-enhancing as well as competence-destroying. Whilst their typology was primarily concerned with capturing competitive, industry-level opportunities and challenges of AI and their higher-level “effects on organizational competencies” (Ibid, p. 147), these challenges and effects also apply to workplaces and workers.

In both product and process innovation, the impact on users and developers of AI may be difficult, if at all possible, to foresee. Detection of primary and, further down the line, secondary effects of AI is typically the hardest during the early implementation stages of an innovation when modifications and adjustments intended to reduce undesirable risks or concrete hazards from the AI innovations may still be comparatively easily applied, and at a lower cost. However, in reality, these risks only become apparent at the stages of testing and final implementation application, when it might be too late or too costly to change direction (Collingridge 1980).

This would indeed appear to be a risk facing many organisations considering the fresh or extended use of AI. Analysis from McKinsey Digital (2020, p. 9) detailing a range of organisational risks of AI found that only “a minority of companies recognize many of the risks of AI use, and fewer are working to reduce the risks”. Cybersecurity inadequacy was the risk most frequently mentioned (62% of surveyed businesses) and also acted upon (with 51% of businesses taking action to mitigate cybersecurity risks in 2020). The second most frequently identified risk was regulatory compliance failure (identified by 48% of businesses, mitigated by 38%). Yet, whilst risks most likely to affect the functioning of the organization as a whole featured strongly, others including personal and individual privacy (39%, 30%), workforce/labour displacement (31%, 19%), equity and fairness (34%, 24%) and physical safety (19%, 15%) received less recognition and drew less of a response. Moreover, the McKinsey survey found that only companies identified as AI leaders were likely to recognise such risks and to implement measures to mitigate them. From a regulatory and risk prevention point of view, this lack of risk awareness amongst organisations, which have yet to develop AI capabilities, is of particular concern.

## AI can optimise workplaces, but also burden and harm workers

The apparent lack of awareness contrasts with some of the emerging evidence of observations and experiences of workers affected by AI, for whom the risks of AI are more than an abstract concern (e.g., Trades Union Congress 2018; Commission on Workers and Technology 2019). In particular, employees have reported that, as a result of the introduction of AI, enjoyable aspects of their work have been removed; that they had little or no say in how business processes or worker roles were changed; that there was less frequent, lower quality communication between employees and employers (e.g., videoconferencing instead of meeting face-to-face, accenting power differentials in team dynamics); that there was downward pressure on wages and conditions (with employers placing a premium on employees’ flexibility); and that there was a disproportionate impact of work displacement on the unskilled and on specific groups such as women and older/younger workers.

The use of sophisticated company ICT systems to specify, allocate, and complete tasks is already broadly associated with an increased level of worker surveillance, enabled via large-scale, high-volume data collection on worker activity (Ajunwa et al. 2017). Increasingly, detailed data on the specifics of worker activity, not just task completion, is available as an on-demand company performance metric, covering not just what workers do, but also how they do it. This enhanced level of worker surveillance has largely been normalized as acceptable in management practice, particularly via the availability of dashboards offering visual overviews of quantitative data on key organisational performance metrics. Such systems permit monitoring of workers from initial system log-on, to an overview of task completion rates, and potentially down to the micro (keystroke or tap) level, including the use of biometric data in some instances.

Over the past few years, AI-enabled technological advances have facilitated deeper integration of these increasingly diverse data sources on workers, including unstructured sources such as speech or video (e.g., natural language processing of recorded telephone calls; facial recognition analysis of webcam data), and have promoted new understanding of worker data through “big data” analysis techniques, such as predictive modelling. Kellogg et al. (2020), in reviewing and summarizing the emerging literature on the topic, explored some of the ways that AI might affect workers in more detail. They suggested that AI systems enabled organisations to direct, evaluate, and discipline workers. In addition to the traditionally more passive oversight of workers offered by company ICT systems, new AI systems actively directed workers by restricting and recommending information or actions, such as generating scripts for call centre staff to use without deviation or specifying travel routes and times that gig workers in the delivery industry are expected to follow and meet. This phenomenon of AI systems, rather than managers, directing workers is now termed “algorithmic management” (Schildt 2017; Lee 2018; Noponen 2019; Jarrahi et al. 2021). It describes employers using AI to evaluate how workers performed tasks and assessed their behavioural patterns, determining which employees were best suited for different tasks.

Whereas management may seek to use AI to optimise workflows and identify diligent, effective employees for the right tasks, AI may also be used to discipline workers who do not meet their criteria of diligence. Kellogg et al. (2020) noted examples of AI having been used to discover erratic and dangerous driving behaviour in taxi drivers or detect safety violations, such as not wearing appropriate safety attire when entering restricted areas. AI applications were here used for positive purposes, but their effects on workers was still, at least occasionally, negative, causing frustration with unintelligible algorithmic “nudges” and unwelcome recommendations due to unexplained communications, such as mandated rest periods. Organisations thus introduce AI systems to track and micro-manage employees through intrusive data collection means (Moore 2019; Mateescu and Nguyen 2019). The integration of monitoring tools into the physical space of a worker, such as a smart wristband, exacerbates health and safety risks where they tend to accelerate the pace of work whilst diminishing workers’ task control (Horton et al. 2018; Moore 2018). Algorithmic management is thus becoming an increasingly familiar form of working in technology and other service companies, and advanced manufacturing businesses alike (Wood 2021). Often hidden from the view of those most directly affected, this practice has been blamed for causing a loss of “dignity and the wherewithal of workers” (Jarrahi et al. 2021, p. 10).

## Gaps and challenges in WHS practices to identify and manage AI risks

The above accounts provide hints as to how introducing AI may affect workplace health and safety (WHS), but a detailed exploration and understanding of their connection is largely absent. WHS—sometimes also referred to as occupational health and safety (OHS)—has been defined as conditions and factors “that affect, or could affect, the health and safety of employees or other workers (including temporary workers), visitors or any other person in the workplace” (BSI 2011, p.1). Internationally, governments, and public and private organisations promote or adopt occupational health and safety management (OHSM) standards such as OHSAS 18,001, or its recent descendent ISO 45001, to guide the implementation of policies and practices which aim to maintain a safe workplace (Jespersen et al. 2016a, b; Jain et al. 2021).

**Fig. 1:**
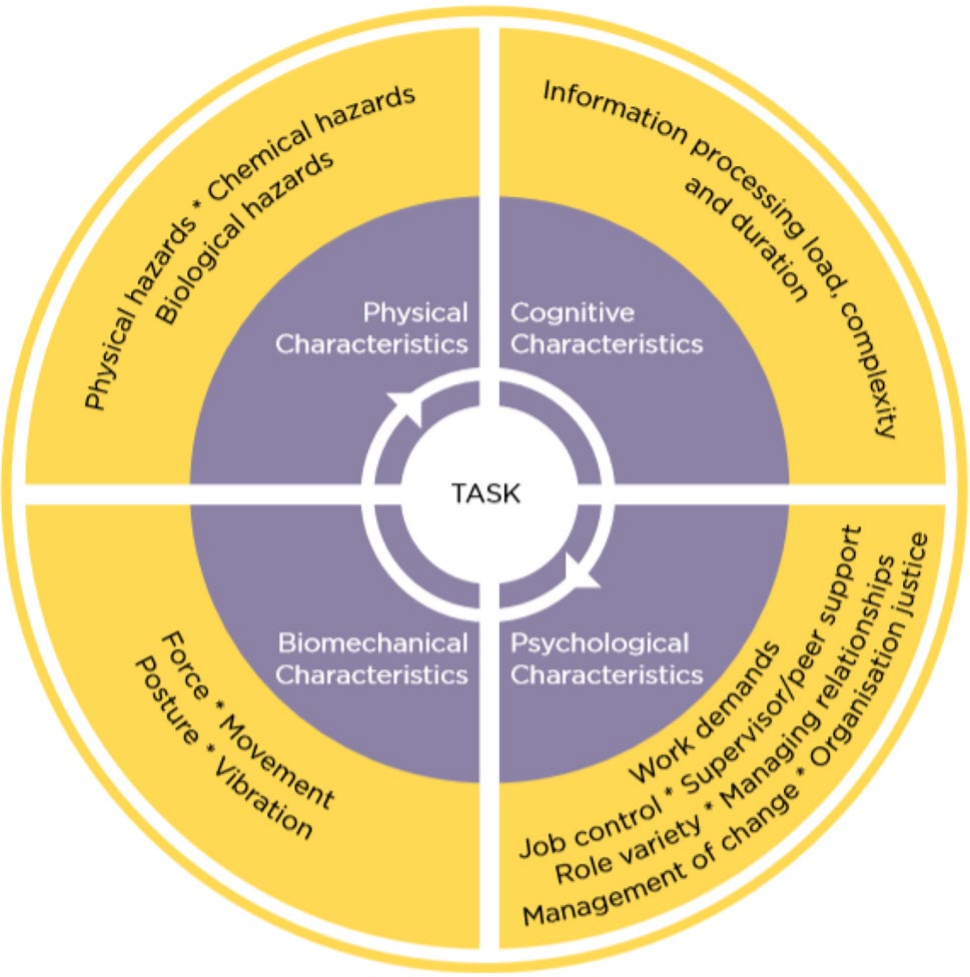
Key characteristics of work. Source: Safe Work Australia 2015, p.9

These standards typically mandate some variation of a “Plan-Do-Check-Act” cycle^1^ (Arntz-Gray 2016) and require internal and external audits which check the degree to which an organisation's implementation meets regulatory requirements (Jespersen et al. 2016a, b). In the Australian regulatory context of WHS, the activities that organisations are expected to observe, monitor and assess are detailed in statutory “Principles of Good Work Design” (Safe Work Australia 2020) as well as the broader legislative framework (e.g., the Work Health and Safety Act 2011). The “Principles” determine that “Good work design addresses physical, biomechanical, cognitive and psychosocial *characteristics of work*, together with the needs and capabilities of the people involved” (ibid, p.9, emphasis added). As can be seen in Fig. 1, which has been taken from the “Principles of Good Work Design”, characteristics of work are specific to work tasks and each is associated with a set of “hazards and risks” as detailed in the outer circle of the figure, which in turn may entail specific, potentially adverse effects for health and safety in the workplace.

WHS is not necessarily concerned with the elimination of all sources of risk in the workplace, but more realistically with promoting a systematic approach to hazard management (Safe Work Australia 2015). Yet, WHS schemes tend to be best suited for addressing situations where there is a straightforward connection between the cause of a specific safety risk and its resolution. For example, to address the risk of an industrial robot colliding with a human, the machine could be placed behind a fenced area. With AI this is changing as the new technology may not solely or indeed primarily pose visible, detectable physical risks and points of hazards, but also and more frequently psycho-social risks resulting from AI’s de-humanising application. This is a challenge for WHS, which tends to favour the regulation of physical safety-related risks but is not so well-suited for scenarios where the risk is ambiguous and its resolution complicated and multi-faceted, as its injury may be psycho-social.

Psychological harm is likely triggered when individuals feel unable to bridge the gap between their capabilities and the requirements or expectations placed on them, but it may also reflect poor work organisation, a command-and-control management style, lack of support for work-life balance, or harassment, bullying, mobbing and verbal abuse (Nieuwenhuijsen et al. 2010; Leka and Houdmont 2010). Because psychosocial considerations involve subjective assessments and are often situation-specific, the WHS standards often fail to address psychosocial risks in practice (Jespersen et al. 2016a, b).

Highly demanding jobs over which an employee has little control are especially prone to give rise to psychological and physical strain but are less likely to do so if and when employees are given greater control of the conduct (timing, sequencing, speed) of their tasks (Karasek 1979; Leka and Houdmont 2010). It has thus been argued that AI solutions should privilege human autonomy, build and encourage competence in the form of feeling able and effective with on-the-job tasks, and enable relatedness in the form of feeling connected with people, especially those involved in on-the-job tasks (Calvo et al. 2020; Ryan and Deci 2017).

## Human dignity and autonomy in the AI using workplace

Granting human autonomy is a critical contribution to making work meaningful (cp. Smids et al. 2020) and to maintaining dignity at work (Bal 2017). At a theoretical and conceptual level, human dignity refers to the intrinsic worth of every human being that distinguishes them from any other creature (Sison et al. 2016). Their privileged social status gives rise to various obligations on how people treat each other. In a workplace context, this has been translated to mean safeguarding equality, contribution, openness, and responsibility (Bal 2017). Practically, it means that employers need to consider the wellbeing of their employees in the context of whatever tasks they assign their workers, offering purpose and ruling out “submission to demeaning or arbitrary authority, unhealthy or unsafe conditions, or physical or mental degradation” (Autor et al. 2020, p. 5).

The workplace here is a social institution because one works in collaboration with people in the service of other people. The workplace and work itself serve as one of the most important social channels through which humans can flourish, and human dignity can manifest (Sayer 2007, 2009). The workplace is not solely a means for us to earn a livelihood, but also allows us to grow and advance in our knowledge, skills, and productive habits. It shapes our attitudes towards our culture and society and helps us appraise the purpose and goals of our life (Sison et al. 2016). When we begin to identify with our work, we bring implicit dignity to it as something personal and meaningful to us.

When considering the adoption of AI in the workplace, it is essential to contemplate how the use of a particular AI system will affect worker’s dignity “in” and “at” work (Bolton 2010). Dignity “in” work signifies work that is interesting and meaningful with a degree of responsible autonomy and recognised social esteem. Dignity “at” work implies workplace structures and practices that offer equality of opportunity, collective and individual voice, safe and healthy working conditions, secure terms of employment and just rewards. A naïve exploration of AI use cases in the workplace risks framing AI-assisted work solely through the lens of its professional significance, and not its human impact.

When an organisation adopts an AI system, it affects not only a worker’s agency, but also the structural and cultural domain of the workplace (Donati 2020). Specifically, when one introduces an AI system into the workplace, it implicitly or explicitly occupies a position in the social structure. It thus changes the social structure of the people who interact with the device. For example, an AI system could take over the role of scheduling work which was previously done by a line manager. The resulting modified social structure influences the worker’s agency (how they act). The worker’s agency, in turn, exerts an influence on the cultural domain and affects the way work is perceived and understood. For example, employees may regard the work that an AI assigns as impersonal and lacking purpose. Social interactions emerge in which the algorithm may take dominance, and the workers must modify their ways of thinking and expressing themselves (Donati 2020). For instance, they may need to learn a new digital literacy to engage with the AI system properly. As workers assimilate an AI’s way of operating the resulting cultural shift may affect how the AI system performs. The potential for a cultural change to impact AI is exceptionally high when the AI is “continually learning” from data. For example, employees may change how they work to optimise whatever objective they perceive the AI system to use when it assigns work. Consequently, the AI system could switch from setting employees’ large contiguous chunks of work to small, fragmented jobs in response to changes in how employees perform their jobs (i.e., the speed with which they complete tasks, etc.). The evolution of the AI system’s operation may adjust its position on the social structure, once again modifying worker’s agency and the whole cycle repeats.

The creation of new workflows through AI can thus involve, perhaps continuous or at least repeated, elements of work redesign for human actors, and potentially, a new conception of teamwork involving AI algorithms/systems as functional team-members (Griffin et al. 2019). In such circumstances, preserving and promoting the dignity of work requires scrutinising the decisions and actions made in the workplace based on AI outputs. Donati (2020) emphasises the need to enhance the capacity of the worker to manage relations with technology through digital literacy and feedback mechanisms. This argues for organisations to reflect on the qualities and causal properties of the human relationships that an AI-augmented job allows or, on the contrary, obstructs or impedes. For example, the degree to which an AI prediction, decision or recommendation is explainable or contestable may have a direct impact on one’s workplace relationships. Donati (2020) also stresses the value of increased awareness of work as a social relation, and not only as functional performance. Viewing work as a social relationship allows one to easily recognise the need for establishing proper oversight and governance structures that monitor the degree to which AI systems simultaneously facilitate self-realisation and professional output.

## AI ethics frameworks

Thinking about AI governance in terms of social relations and developing appropriate workplace oversight structures that align with Human Factors principles is facilitated by referencing already established ethical guidelines for AI. Various ethical guidelines for AI have been developed around the world, be they led by governments (e.g., Australia, Canada, and Singapore) and the industry (e.g., Microsoft, Google, Open Data Institute). Hagendorff (2020) identifies 22 examples of such AI ethics guidelines; however, the legal and regulatory status of these guidelines differs by jurisdiction, although in the main, their adoption is optional. This raises the question of the overall effectiveness and impact of such guidelines, leading Hagendorff to assert that AI ethics “lacks mechanisms to reinforce its own normative claims” (Hagendorff 2020, p. 99). In Australia, CSIRO/data61’s Strategic Insight team, in partnership with the Australian Government Department for Innovation, Industry and Science, and the Office of the Queensland Chief Entrepreneur, led a project to develop an AI Roadmap and Ethics Framework under the banner of “Building Australia’s artificial intelligence capability”. The Framework was published in April 2019 (Dawson et al. 2019) and has subsequently been adopted by Federal Government (DISER undated) and State governments, including the NSW Government (NSW Government 2019). Table 1 list the eight ethics principles, categorised into three higher-level aggregates that span the human condition, worker safety and oversight.

**Table 1:** Higher-level aggregates of the AI ethics principles

Human condition	Worker safety	Oversight
**Human, social and environmental wellbeing**: Throughout their lifecycle, AI systems should benefit individuals, society and the environment **Human-centred values**: Throughout their lifecycle, AI systems should respect human rights, diversity, and the autonomy of individuals **Fairness**: Throughout their lifecycle, AI systems should be inclusive and accessible, and should not involve or result in unfair discrimination against individuals, communities or groups	**Privacy protection and security**: Throughout their lifecycle, AI systems should respect and uphold privacy rights and data protection, and ensure the security of data **Reliability and safety**: Throughout their lifecycle, AI systems should reliably operate in accordance with their intended purpose	**Transparency and explainability**: There should be transparency and responsible disclosure to ensure people know when they are being significantly impacted by an AI system and can find out when an AI system is engaging with them **Contestability**: When an AI system significantly impacts a person, community, group or environment, there should be a timely process to allow people to challenge the use or output of the AI system **Accountability**: Those responsible for the different phases of the AI system lifecycle should be identifiable and accountable for the outcomes of the AI systems, and human oversight of AI systems should be enabled

Source: Authors, based on DISER (undated)

The CSIRO/data61 AI ethics framework helps to explore the extent to which AI may affect individual wellbeing, values and rights, but the ethical principles at its core are abstract, and in their current form, not amenable to use for assessing workplace safety and workforce wellbeing concretely. Ethics principles provide a values-based framework for human-centred AI development, but no guidance to organisations on how to operationalise these principles. To apply ethics principles to everyday use in a workplace context, a tool is required to connect these principles to the realities of workplaces, their processes and innovations. The tool adopted in this applied research exercise was the AI Canvas (Agrawal et al. 2018a).

## The AI Canvas

The AI Canvas is a decision support tool for businesses and organisations considering implementing AI. Developed by a team of researchers at the University of Toronto, its purpose is to help business leaders and managers to understand whether adopting AI will enable them to achieve their strategic goals.

Agrawal et al. (2018b, p. 140) emphasise that the Canvas is a tool to aid with decomposing tasks within a workflow, “in order to see where prediction machines can be inserted”. This act of “decomposition” then enables a return-on-investment analysis and ranking of the various opportunities to apply AI. For this study, the AI Canvas served as a tool for dissecting the process of AI adoption into ideal–typical stages during which AI ethical and WHS risks may arise. The AI Canvas proposes a set of seven categories of questions that decision-makers would need to consider in determining whether adopting AI will advance their overall organisational or operational strategy.

The seven categories and associated questions are:

1. Prediction: what does the AI need to predict?
2. Judgment: how do we value correct versus incorrect predictions?
3. Action: how do the predictions affect what we do?
4. Outcome: how do we measure the performance of the AI?
5. Input: what data does the AI need for deployment?
6. Training: what information does the AI need for training?
7. Feedback: how can we use outcomes to improve the AI continually?

The AI Canvas in its current form is highly technocentric. It is focused on the potential of a proposed AI system and its technical underpinnings from a commercial point of view and encourages thinking about AI through a narrow lens of financial concerns. The AI Canvas is less able to evaluate the organisational and human relations context in which an AI system is deployed, maintained, and eventually, decommissioned. Just as the AI Ethics Principles lack immediate application to work contexts, the AI Canvas lacks direct reference to the fundamental question as to whether a proposed AI system is ethical, human-centred, and in alignment with organisational and societal values.

For this study, connecting the AI Canvas and AI Ethics Principles was a first step towards addressing their respective weaknesses with respect to practically understanding and identifying the health and safety aspects of AI use in the workplace. Further adding the Principles of Good Work Design (discussed earlier, Fig. 1) enables reframing of the ethical concerns as WHS risks and hazards, thus, contextualizes AI ethics principles in a workplace environment. This helps to identify specific sources and types of risks and hazards in the workplace, but importantly it leads to awareness and stimulates reflection on effects that AI may have on workers’ health and safety.

## Conceptual integration of AI adoption and WHS viewpoints

**Fig. 2:**
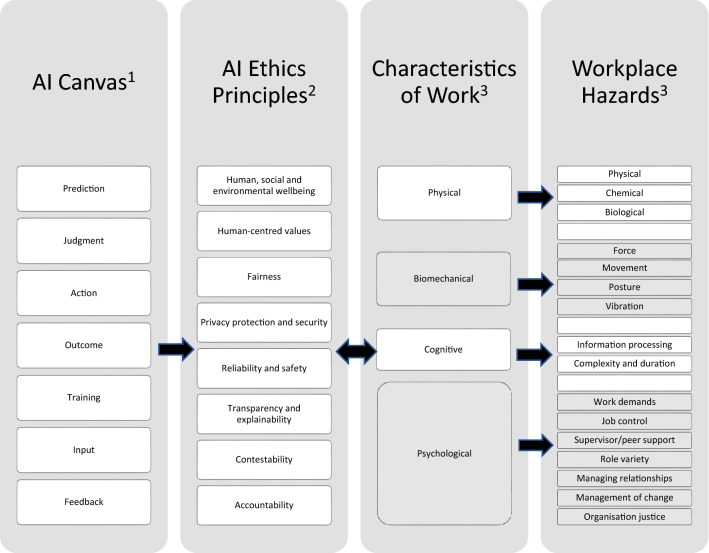
Conceptual integration of AI Canvas, AI ethics principles and safe work characteristics. Sources: ^1^Agrawal et al. (2018a); ^2^DISER (undated); ^3^Safe Work Australia (2015)

Figure 2 illustrates the concept of our incorporation of the AI Canvas via the AI Ethics Principles to SWA’s ‘Principles of Good Work Design’ schemata, thus connecting higher level, abstract ethical concerns, with characteristics of work and their related concrete risk and hazard. Making these connections sequentially resulted in the AI Canvas being cross-tabulated with the AI Ethics Principles, which together were then populated with AI-specific WHS risks and hazards.

The connections were established empirically through a practice led qualitative approach, incorporated by literature reviews and a series of consultations with AI experts, WHS professionals, regulators and policymakers, representatives from organisations adopting or having adopted AI, and others with expertise in the field. Initially, a total of 30 interviews (26 AI experts, 4 WHS experts) and 2 public online workshops with 22 participants were conducted to capture their experience of adopting AI in the workplace, and their perceived awareness of ethical AI in relation to WHS; and to explore healthy and safe use of AI in the workplace in identifying and managing its WHS risks. The consultations, along with the review of the literature, were critical for establishing an initial matrix with appropriate AI-risk relevant content. A second round of interviews was conducted with 12 individuals from 9 different organisations (including 4 AI users in public and private sector, and 5 AI advocates or vendors) to understand the AI adoption process in practice and the WHS management around the use of AI in the workplace. These business focused consultations also contributed to the refinement of the WHS risk matrix. Further consultations with 15 WHS professionals, via an online workshop, confirmed the relationship between AI ethical risks and the SWA schemata. Throughout great emphasis was placed on focussing the conversations on matters related to workplaces, their operations and the relationships that they sustained. This was necessary as, except for some organisations adopting AI, for many experts and practitioners the ethical and safe use of AI in a workplace (other than the platform economy) had previously rarely been reflected upon.

Moreover, although there is a rich literature on the generic topic of AI ethics, as noted earlier, there is very little that specifically addresses what it concretely means to apply AI ethically and safely in the workplace. To be informative to the present exercise, the literature review drew only on those items, which specifically addressed AI matters relevant or, better, directly relating to workplaces. In many instances, this required making associations that the literature might not have discussed in a workplace context but which, based on reasonable interpretation, nonetheless bore direct relations to workplaces. An example of this is Amodei et al.’s (2016) deep and complex discussion of the “Concrete Problems of AI Safety”, which addresses the problem of the safe use of AI from multiple dimensions, including oversight, unintended consequences, reward hacking, but does so without directly referring to workplaces. Yet many of the risks the authors identified can be related more or less directly to the specifics of safety in offices or on factory floors. Another example is the “Ethical Principles for Technology” developed by the Sydney-based independent think-tank, The Ethics Centre (Beard and Longstaff 2018). The Centre approaches the challenge of ‘good AI’ from a philosophical angle, which it applies through product examples and brief case studies to actual uses, albeit outside the workplace.

In other instances, associations to the workplace were more apparent, such as in WHS safety research undertaken on the use of robot/robotics in the workplace (e.g., TNO undated). Besides raising issues of the *physically safe* deployment of robotic equipment, the TNO (undated) study also relates questions of workplace management and task control, factors increasingly infringed upon by AI. Another example is the ‘risk chain’ in AI services proposed by Matsomoto and Ema (2020) who apply their model to a constructed case of a “personnel department in Company A”, which uses AI to predict and select recruits based on their job applications they submit for vacancies in the organisation. Yet Matsomoto and Ema also only refer to conventional, narrow concepts of fairness, robustness, explainability and “proper use” as conceptual tools for assessing AI risk. The risk of AI is then defined as the tool’s ability to correctly identify recruits a priori, but without paying regard to the effects that a de-humanised recruitment process might have on the new colleagues thus recruited (or those *not* recruited), the existing workforce, or recruiters and their task control.

The overview of the state of research on the use of algorithms to plan and organise workloads and work patterns by Kellogg et al. (2020), noted earlier in this paper, arguably remains the most analytical and systematic contribution to this literature. To this we may add a recent empirical account of gig work in the food delivery economy in Australia by Convery et al. (2020). In the absence of any further, robust empirical accounts of AI risks and hazards *directly* relating to workplaces, the present study turned to the secondary literature to conceptualise and apply WHS risks and hazards described in the context of robotics, computing and consumer risks of AI to corresponding workplace scenarios. This was achieved by extracting relevant concepts, ideas and generic examples of risks and hazards and to apply these to the matrix combining AI Ethics Principles and the AI Canvas. To populate the AI WHS scorecard’s matrix, this research adopted a conceptual human dignity-human autonomy model, which applied Bal’s (2017) dignity of work concept as it encompasses equality, contribution, openness, and responsibility; alongside Donati’s (2020) concern for the effective management of autonomy within a socially relational setting that engages with technology through joint competence building and responsible governance. For each matrix cell, the questions asked were:

- How might the combination of AI ethics principles and AI canvas stages affect equality, contribution, openness, and responsibility in a workplace using AI; and
- What measures might be missing but ought to be available as their absence could undermine the responsible, competent governance of human-AI relations in a workplace?

Table 2 identifies risks and hazards thus identified for each of the matrix cells, including references to literature that identified or informed our specification of the named risks or hazards. The specification of risks and hazards was undertaken concurrently with the identification of case examples, as illustrated in the penultimate column of Table 2. Although the research identified examples for each risk or hazard, for space reasons, those reproduced in Table 2 are limited to the ‘human condition’ AI Ethics Principle (first column) as it may be impacted in the AI Canvas stage referenced in the same row as the example. The exception is the AI Canvas ‘feedback’ example, which relates to the worker safety AI Ethics Principles as no ‘human condition’ AI Ethics Principle was identified as potentially affected. The list of risk specifications and associated examples continues to be developed and may not be final until this field of research has a much more complete understanding of the currently unforeseen and unforeseeable workplace effects of AI than it has at present.

**Table 2:** Revised AI WHS Scorecard with examples of AI WHS risks identified in the literature and the workshops

Main stages of development	AI Canvas	AI WHS Principles									Examples*	Safework Characteristics of work & hazards/risks
		Human condition			Worker safety			Oversight				
		Human, social and environmental wellbeing	Human-centred values	Fairness		Privacy protection and security	Reliability and safety	Transparency and explainability	Contestability	Accountability		
Ideation	**Prediction**: Identify the key uncertainty that you would like to resolve	• Using AI when an alternative solution may be more appropriate or humane. [5,12] • The system displacing rather than augmenting human decisions. [3] • Augmenting or displacing human decisions with differential impact on workers who are directly or indirectly affected. [7,9,13] • The resolution of uncertainty affecting ethical, moral or social principles. [9,11,14]			• Overconfidence in or overreliance on AI system, resulting in loss of/diminished due diligence. [3,7]			• Inadequate or no specification and/or communication of purpose for AI use/an identified AI solution. [2,7,9,15,16]			Predicting a worker's physical or mental exhaustion levels for monitoring purposes without instituting strategies to prevent exhaustion in the future (worker safety)	Psychological—Work demands
	**Judgement**: Determine the payoffs to being right versus being wrong. Consider both false positives and false negatives	• (Insufficient consideration given to) unintended consequences of false negatives and false positive. [2,4,11,12] • AI being used out of scope. [3,4,7] • AI undermining company core values and societal expectations. [5,14] • AI system undermining human capabilities. [5] • trading off the personal flourishing (intrinsic value) in favour of organisational gain (instrumental good). [14]			• Technical failure, human error, financial failure, security breach, data loss, injury, industrial accident/disaster. [1,7,16] • Impacting on other processes or essential services affecting workflow or working conditions. [1,13]			• Insufficient/ineffective transparency, contestability and accountability at the design stage and throughout the development process. [12,16]			False negatives or false positive disadvantage or victimise a worker, causing stress, overwork, ergonomic risks, anxiety, boredom, fatigue and burnout, potentially building barriers between people, facilitating harassment or bullying (human condition)	Psychological- Work demands
	**Action**: What are the actions that can be chosen?	• Inequitable or burdensome treatment of workers. [1,10] • gaming (reward hacking) of AI system undermining workplace relations. [4,16] • Worker attributing intelligence or empathy to AI system greater than appropriate.[3] • Context stripping from communication between employees.[3] • Worker manipulation or exploitation. [5,7] • Undue reliance on AI decisions. [3,7]			• Adversely affecting worker or general rights (to a safe workplace/physical integrity, pay at right rate/EA, adherence to National Employment Standards, privacy). [1,7] • Unnecessary harm, avoidable death or disabling injury/ergonomics. [1,7,8,16] • Physical and psychosocial hazards. [3,16]			• Inadequate or closed chain of accountability, reporting and governance structure for AI ethics within the organisation, with limited or no scope for review. [7,10,14] • (lack of process) for triggering human oversight or checks and balances, so that algorithmic decisions cannot be challenged, contested, or improved. [3,9] • AI shifting responsibility outside existing managerial or company protocols, and channels of internal accountability (via out- or sub-contracting). [13]			A workflow management system disproportionately, repeatedly or persistently assigns some workers to challenging tasks that others with principally identical roles can thus avoid (human condition)	Cognitive-Complexity and duration
Development	**Outcome**: Choose the measure of performance that you want to use to judge whether you are achieving your outcomes	• Chosen outcome measure not aligning with healthy/collegial workplace dynamics. [1,7] • Outcome measure resulting in worker-AI interface adversely affecting the status of a worker/workers in the workplace. [3]			• Performance measures differentially and/or adversely affecting work tasks and processes. [2,6,10]			• Workers (not) able to access and/or modify factors driving the outcomes of decisions. [2,3,9,16]			Efficiency improvements have differential effects across the workforce, improving conditions for some, but not others, or creating or promoting competitive behaviours, undermining collaborations or collegial relations (human condition, worker safety)	Psychological- Organisation justice
	**Training**: What data do you need on past inputs, actions and outcomes to train your AI to generate better predictions?	• Training data not representing the target domain in the workplace. [7,15] • Acquisition, collection and analysis of data revealing (confidential) information out of scope of the project. [7] • data not being fit for purpose [5,8,11,16]			• Cyber security vulnerability. [1,11] • (In)sufficient consideration given to interconnectivity/ interoperability of AI systems. [2,9]			• Inadequate data logs (inputs/outputs of the AI) or data narratives (mapping origins and lineage of data), adversely affecting ability to conduct data audits or routine M&E. [7,9,10,12] • (Rapid AI introduction resulting in) inadequate testing of AI in a production environment and/or for impact on different (target) populations. [2,4]			Training data for a new system of leave and sick leave projections include only more recent workplace recruits with shorter tenure for whom better contextual data are available (human condition)	Psychological- Organisation justice
	**Input**: What data do you need to generate predictions once you have an AI algorithm trained?	• Discontinuity of service. [1,13] • Worker unable or unwilling to provide or permit data to be used as input to the AI. [9,15]			• Impacting on physical workplace (lay out, design, environmental conditions: temperature, humidity). [10,15] • (In)secure data storage and cyber security vulnerability. [1,2,7,10,16] • Worker competences and skills (not) meeting AI requirements. [13] • Boundary creep: data collection (not) ceasing outside the workplace. [8,15]			• Insufficient worker understanding of safety culture and safe behaviours applied to data and data processes within AI. [8,13] • Partial disclosure or audit of data uses (e.g., due to commercial considerations, proprietary knowledge). [14,15]			A workforce planning tool omits timely correction for seasonal factors, trends or shocks, leading to a shortage of staff or produce at key times (human condition)	Cognitive- Complexity and duration
Application	**Feedback**: How can you use the outcomes to improve the algorithm?				• Assessment processes requiring review due to new approach or tool. [9] • Identifiable personal data retained longer than necessary for the purpose it was collected and/or processed. [10]			• Inadequate integration of AI operational management into routine maintenance ensuring AI continues to work as initially specified. [3,4,8,16] • No offline systems or processes in place to test and review veracity of AI predictions/decisions. [9]			A new HR recruitment process using AI achieves a more gender-balanced intake of new staff. Do the data input or algorithm require review to maintain this outcome? (worker safety)	Cognitive—Psychological, Information processing load, complexity and duration, organisation justice

Legend: Numbered citations refer to the following sources

*Examples pertain to human conditions ethics principle (first Column) and the AI Canvas item in the same row. For the AI Canvas ‘Feedback’ item, the example relates to the worker safety ethics principle

[1] ADAPT Centre et al. (2017); [2] AiGlobal (undated); [3] Amodei et al. (2016); [4] Beard and Longstaff (2018); [5] IEEE (undated); [6] Matsumoto and Ema (2020); [7] ODI (2019); [8] TNO (undated); [9] UK Cabinet Office (2020); [10] van de Poel (2016); [11] Walmsley (2020); [12] WEF (2020); [13] Wikipedia. (2020); [14] Public online Workshop

The generation of the list and the associated examples continued in an iterative process of investigation and discussion amongst the research team and reflective explorations and testing of assumptions with AI and WHS experts, including in businesses that had implemented or were about to implement AI in their workplaces. The discussions with experts added a few more examples of risks, but no new risks as such. The central insight gained from AI experts and business users of AI pertained to the process of consultation within the organisation that used or considered the use of AI. Employee engagement was understood to increase and have increased anticipation and awareness of potential direct and indirect impacts of AI use on workforces. However, it did not safeguard entirely against later challenges as organisational practices and processes in business areas several steps removed from where AI was being used were affected, calling for adjustments going further than originally anticipated.

Employee engagement was also seen to provide the means to connect the language and discipline-specific technical understandings of AI experts, WHS professionals and (other) employees. Building a mutual understanding of the aims, objectives, operating principles and outcomes of AI was thought to be essential for its effective, competent use. Understanding how AI could impact different roles and occupations was argued to be critical for anticipating risks and ensuring AI systems were safe.

These considerations flowed into the design of the AI WHS scorecard as it adopted a stepwise approach to identifying stages in the conceptualisation, design and application of AI. Organisations could benchmark their practices against each of the AI ethics principles, themselves linked to the more likely familiar SWA risk and hazard schemata (for more details, see Cebulla et al. 2021). That final connection to current SWA workplace hazards and risks concepts and terminology is illustrated by the final column in Table 2. AI risks initially defined as ethical for analytical reasons are thus shown to relate to and be accommodated within existing SWA frameworks, although they do need explicit articulation in a framework that currently does not capture sufficiently the complexity of an AI supported workplace.

The utility of the scorecard is best understood in the context of concrete examples. Consider an organisation that uses various machinery and equipment while delivering its service to customers. It struggles with unplanned downtime costs due to sporadic equipment failure. The interruptions result in revenue loss, component replacement costs, and even fines for not delivering its service. Currently, the organisation uses a time-based maintenance schedule where a piece of equipment gets maintained and serviced at fixed time intervals whether it needs it or not. The time-based maintenance is labour intensive and ineffective in identifying problems between the scheduled inspections. The organisation wants to address this problem by adopting AI for predictive maintenance. Our Technical Report^2^ (Cebulla et al. 2021) includes supplementary material describing how one can use the scorecard in Table 2 to help this organisation identify potential AI-based workplace health and safety issues.

## Conclusion

A growing use of AI will change economies, the manufacturing of goods and delivery of services. It will also affect human relations at a societal level, including how we collect, use fairly and keep safe the data that drive AI. Whilst much of the debate of the ethical use of AI has focussed on the societal level, AI also has profound implications in and for the workplaces where it is being used. These workplaces are currently and in most industrial and late-industrial nations regulated by WHS guidelines, rules and prescriptions, which rarely address the specific impact of AI on processes, job roles, and communication among employees, and between employees and managers.

In this paper, we have outlined a model for detecting risks and hazards that AI may bring to workplaces, using a set of AI Ethics Principles, as endorsed by the Australian Government, and the AI Canvas, a technical stages model of AI implementation. The explorative process commenced with a review of the literature on AI risks and hazards as they may apply to workplaces. These potential risks and hazards were further explored in consultation with AI experts, professionals and users—a process that also helped to populate the risk assessment matrix with examples of workplace specific AI risks and hazards. These risk examples were further developed and validated during consultations with AI adopters and WHS professionals. Each of these risks and hazards was linked to statutory WHS principles as advocated by SWA, the Australian WHS regulators and oversight agency.

The language of WHS is already familiar within organisations, which may help organisational leaders as they grapple with the task of promoting a shared vision of what it means to be a safe and healthy AI-adopting workplace. This is however not to underestimate the organisational, political, and regulatory challenges that a safe and ethical adoption of AI faces in present day “hypercommercialized capitalism” (Milanovic 2019, p. 176). The realisation of human-centered AI in a workplace faces barriers, ranging from an overhyped acceptance and exploitative utilisation of AI in business, government, and media (Bridle 2018; Zuboff 2019; Eubanks 2018); a labour movement un(der)prepared to articulate concern or form resistance (Nissim and Simon 2021); to human resource management (HRM) systems focussed on employee culpability over corporate accountability (e.g., Chuang and Graham 2018).

To be effective, HRM will need to evolve to meet the AI challenge successfully. Its WHS principles and policy will need to innovate and thus ‘grow’ to take explicit account of the impact that AI may—and some argue—will have on human–human and human–machine relations in the workplace. This is unlikely to occur in the absence of oversight. The risks of AI system failure or dysfunction may increasingly be well understood by managers with respect to physical hazards affecting workers, such those associated with manufacturing plants, logistics, and customer order fulfilment. Yet as AI—or rather: the acceptance of AI in workplaces—changes who is in control of work processes and who decides the task divisions between humans and between humans and machines, WHS will need to pay more attention to the psycho-social as well as physical conditions shaping workplaces and affecting workers. It is this psycho-social dimension of AI risk that stands most in need of education and advocacy by those responsible for worker well-being, and those with an interest in developing human and organisational potential in the age of AI.

In designing the risk matrix and the more elaborate scorecard, our intention and moderate ambition was to offer an educational and analytical tool for illustrating how WHS risks may come about. The tool should help to anticipate WHS risks whilst modifications to AI implementation and usage plans are still feasible and affordable to avoid unnecessary harm to workers. The scorecard is in a conceptual form at this stage, expected to be further developed and disseminated for implementation as a practical tool, with uptake from organisations that use or intend to use AI in the workplace.

Looking ahead, one of the arguable shortcomings of our study was the absence of a detailed explorations of the conditions beneficial if not critical to the successful application of an AI WHS Scorecard – and of the initial risk matrix (Table 2). Future research may wish to explore how AI adopters may be encouraged to reflect proactively about AI-induced changes in the workplace, and how AI related risks may be anticipated to be minimised, if not prevented from occurring altogether.

Furthermore, whilst our work so far has identified AI WHS risks and hazards, further work is needed to explore strategies for preventing, responding to or ameliorating risks and hazards, which the scorecard has identified.

Finally, it is important to acknowledge that the landscape of AI WHS risks and hazards is likely to be continuously evolving alongside the maturing of AI technologies and the emergence of a more diverse universe of public, private, and third sector adopters. This growth presents both an opportunity and a challenge. First, an opportunity to gather more data and insights on potential and emerging AI WHS risks and hazards, and to catalogue and assess these. Second, a challenge to devise protective measures (instruments, policies, and processes) that are cognizant of adopters’ likely varying capabilities of application.

## Data Availability

De-identified interview summaries are available upon request.

## Footnotes

1 Plan: Establish the objectives and processes necessary to deliver results following the organisation’s OHS policy. Do: Implement the process. Check: Monitor and measure processes against OHS policy, objectives, legal and other requirements and report the results. Act: Take actions to improve OHS performance continuously.

2 The Technical Report of this study, including the AI WHS Scorecard, can be downloaded here: https://www.centreforwhs.nsw.gov.au/knowledge-hub/ethical-use-of-artificial-intelligence-in-the-workplace-final-report. The report and a version of the AI WHS scorecard developed in Excel format with risk scoring content can also be obtained from the authors upon request.
